# Dr. Bonwill’s Visit to Europe

**Published:** 1897-05

**Authors:** 


### DR. BONWILL'S VISIT TO EUROPE.

Dr. Bonwill will attend the International Medical Congress to be held in Moscow, August next, and requests us to say that he will *en route*, going and returning, be pleased to meet, at any of the stop-over cities, the members of the dental profession, and either give clinics or talk on any of the subjects with which he is familiar. He expects to be in Rome, Florence, Venice, Berlin, Leipzig, Vienna, St. Petersburg, and other places.

